# Constrained α-Helical Peptides as Inhibitors of Protein-Protein and Protein-DNA Interactions

**DOI:** 10.3390/biomedicines6040118

**Published:** 2018-12-18

**Authors:** Siddhartha Roy, Piya Ghosh, Israr Ahmed, Madhumita Chakraborty, Gitashri Naiya, Basusree Ghosh

**Affiliations:** Department of Biophysics, Bose Institute, P1/12 CIT Scheme VII M, Kolkata 700054, India; piyapc@gmail.com (P.G.); israrkolkata@gmail.com (I.A.); jinia20022002@yahoo.com (M.C.); gitashrinaiya@gmail.com (G.N.); basusreeghosh@gmail.com (B.G.)

**Keywords:** helix, synthetic transcription factor, peptide

## Abstract

Intracellular regulatory pathways are replete with protein-protein and protein-DNA interactions, offering attractive targets for therapeutic interventions. So far, most drugs are targeted toward enzymes and extracellular receptors. Protein-protein and protein-DNA interactions have long been considered as “undruggable”. Protein-DNA interactions, in particular, present a difficult challenge due to the repetitive nature of the B-DNA. Recent studies have provided several breakthroughs; however, a design methodology for these classes of inhibitors is still at its infancy. A dominant motif of these macromolecular interactions is an α-helix, raising possibilities that an appropriate conformationally-constrained α-helical peptide may specifically disrupt these interactions. Several methods for conformationally constraining peptides to the α-helical conformation have been developed, including stapling, covalent surrogates of hydrogen bonds and incorporation of unnatural amino acids that restrict the conformational space of the peptide. We will discuss these methods and several case studies where constrained α-helices have been used as building blocks for appropriate molecules. Unlike small molecules, the delivery of these short peptides to their targets is not straightforward as they may possess unfavorable cell penetration and ADME properties. Several methods have been developed in recent times to overcome some of these problems. We will discuss these issues and the prospects of this class of molecules as drugs.

## 1. Introduction

Protein-Protein (PPI) and Protein-DNA (PDI) interactions are one of the most fundamental interactions in the biological system. They are essential components of core cellular processes such as transcription, translation, replication, etc. Dysregulated signaling pathways are often signatures of many diseases. The specific inhibition of a particular PPI/PDI in the dysregulated pathway may lead to the correction of aberrantly regulated pathways in a cell [1,2]. However, inhibition of these interactions with small molecules presents a challenge, particularly against those interaction interfaces that are extensive, shallow, and hydrophilic [3]. An antibody may be useful for the development of protein-based inhibitors of PPIs. Antibodies can be engineered to bind with high affinity to many targets [4]. However, antibodies are currently restricted for extracellular applications. In addition, due to their large size, antibodies are not ideally suited against some forms of solid tumors that require deep penetration. Due to their smaller size and the possibilities of intracellular targeting, peptides hold promise for inhibiting PPIs and PDIs within the cell [5]. 

A large number of protein-protein and protein-DNA interactions responsible for the maintenance of complex gene regulation patterns involve the participation of a common secondary structure element, the α-helix [2,6]. Thus, short α-helical peptides may be effective functional mimics of proteins involved in such interactions and capable of effectively inhibiting PPIs or PDIs. However, once a short unmodified peptide is synthesized in isolation, much of its ability to bind specific biomolecules is weakened, as it tends to adopt random-coil conformations rather than the structured and biologically relevant conformation. Also, another major obstacle in the use of unmodified peptides (due to their extended random-coil conformations) is their inherent susceptibility to proteases [7]. In principle, short helical peptides should be more resistant to proteolysis than their random-coil counterparts, because the proteases generally bind their substrates in an extended conformation. For these reasons, the stabilization of short peptide segments into helical conformations or the development of non-natural scaffolds which mimic helices, have been the center of much focus and attention [8]. Significant progress has been made in the development of α-helix mimetic peptides for potential therapeutic applications [9]. Various strategies that have been employed for constraining α-helices include salt bridges between charged amino acid side chain residues [10,11], lactam bridges [12,13], disulfide bridges [14], hydrogen bond surrogates [15,16], hydrophobic interactions [17], metal ligation [18,19], triazole staples synthesized from alkenyl and azido side chain residues [20], photo-controllable macrocycles [21], introduction of α,α-disubstituted amino acids [22,23] and hydrocarbon staples [24]. Some of the methods for helical peptide stabilization, mentioned above, have been discussed in the following sections. In this article, PDI inhibitors are given wider coverage as PPI inhibitors are more widely covered in the existing literature. 

## 2. Important Elements of Peptide-Based PPI and PDI Inhibitors

**Figure 1:**
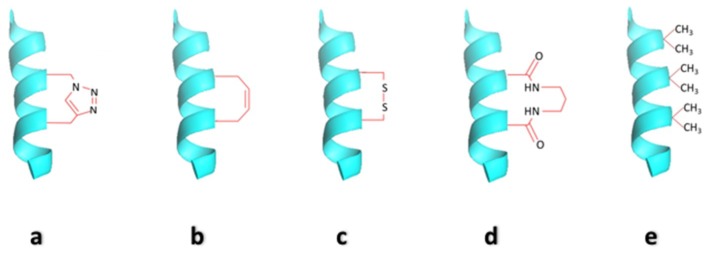
Different α-helix-stabilization methods for small peptides. For cross-linked peptides, all are between residues i and (i + 4). (**a**) Triazole stapled helix; (**b**) Hydrocarbon stapled helix; (**c**) di-sulfide cross-linked helix; (**d**) Lactam cross-linked helix; (**e**) Aib substituted helix.

Work performed in the late 1990s and early 2000s first indicated that peptides with helical conformational preferences might be used to disrupt protein-protein interaction [25,26]. However, these peptides were largely targeted toward extracellular receptors; even when the target was an intracellular protein, no significant attempt was made to study inhibitions of protein-protein interactions inside a cell [27,28,29,30]. The first attempts to inhibit protein-protein interactions using non-peptidic molecules that conformationally mimic α-helices were made by Hamilton and co-workers during the early years of the new millennium. His group developed the terphenyl scaffold as a helix mimetic [31,32]. The use of stabilized peptides for protein-protein interaction inhibition was attempted a few years later through the incorporation of unnatural amino acids or chemical modification of the peptide chain. The actual chemistry of putting conformational constraints is given below in further details and is shown in Figure 1. 

### 2.1. Incorporation of Non-Protein Amino Acids

A large proportion of protein-protein interaction inhibitors have been reported to be α-helices (35). Hence, particular emphasis has been placed on the design of conformational constraints that can induce α-helical structure in short stretches of amino acids. Introduction of many non-protein amino acids, such as the α-amino *iso*-butyric acid (Aib) and other α-methyl amino acids have been shown to increase the helicity of short peptides by a considerable amount without regard to other amino acid types present [23,33,34]. It has been established that oligomers of Aib tend to adopt a 3_10_ helical conformation, whereas peptides in which Aib is incorporated, along with other residues, have a greater tendency to adopt proper α-helical conformations [35,36]. Moreover, substitution of non-interacting amino acids of a short helix by Aib effectively increases the helical propensity of the short peptide, creating a mimic of the targeted protein [27]. This method of constraining peptides to α-helical conformation can be implemented in a straightforward manner by solid phase peptide synthesis using the standard Fmoc chemistry [37].

### 2.2. Side-Chain Cross-Linked α-Helices

The α-helix contains 3.6 residues per complete turn, which places the i, (i + 4), (i + 7), and (i + 11) amino acid side chains on the same face of the helix. The classical strategy to stabilize the α-helical conformation in peptides employs covalent bond formation between either i and (i + 4) or i and (i + 7) side chain groups [38]. The first side-chain cross-linked peptides contained lactam, disulfide and metal-mediated bridges [13,14,39,40]. Helices containing lactam bridges and disulfide links have been successful in targeting their intended extracellular receptors [28,29,41]. Side chain macrolactam formations between i and (i + 4) residues were used to generate α-helical Rev-like peptides which were shown to be an effective inhibitor of the HIV-1 Rev-RRE interaction [42]. 

### 2.3. Hydrocarbon Stapled Helices

Among methods of developing short stabilized helical peptides using linkage between i and (i + 4) and/or (i + 7), hydrocarbon-stapled helices are of prime importance. The stapled helices not only stabilized the helical conformation of the small peptide, but also facilitated cell entry. Grubbs and coworkers showed that olefin metathesis can be used to promote helicity [43]. In 2000, Verdine and coworkers first designed and developed hydrocarbon-stapled helices by a careful examination of linker lengths and stereochemistry. The peptides were stabilized via olefin linkers between positions i and (i + 4) or i and (i + 7). These hydrocarbon-stapled peptides were found to be a marked improvement over their predecessors in terms of structure, potency, protease stability and also, quite surprisingly, in cell penetration abilities [24]. In 2004, Walensky, Verdine, and Korsmeyer published their results on the effects of stabilizing pro-apoptotic BH3-mimetic peptides [44]. Stapled helical peptides were also used to target BCL2 in cell culture and animal models [44]. An (i, i + 7) stapled peptide was designed by Verdine et al., to inhibit the p53-hDM2 interaction and re-establish the pro-apoptotic p53 pathway [5]. Such stapled helices were also used by Zhang et al., to target HIV-1 capsid assembly [45].

Both the lactam-bridged and the hydrocarbon stapled helices may feature flexible crosslinks. From entropic considerations, rigid linkers might afford more stability to constrained helices. Two groups have recently studied the effect of linker flexibility on helix stability. Woolley and co-workers found that a rigid aromatic linker that matches the distance between i and (i + 11) side chains provides greater stability than a flexible linker [45]. Fujimoto et al., have reported a detailed study of various flexible and rigid linkers, and crosslinking positions on the helix [46]. They have hypothesized and demonstrated that rigid linkers which are shorter than the target helix pitch, lead to more stable helices. These interesting findings may lead to the re-evaluation of the lengths of linkers used in side-chain cross-linked helices [38].

### 2.4. Cysteine Bis-Alkylation for Helix Stabilization

A variety of cysteine alkylation strategies can be employed for generating different conformationally constrained peptides. Different cysteine alkylation reactions are briefly described in this section. One of the reactions is a thioether ligation between cysteine and bromoacetylated ornithine, yielding an alternative to lactam bridges [47]. Recently, a thiol-based alternative to ring-closing metathesis reaction was used to staple two cysteine residues in peptides with a diene linker [48]. Two or three unprotected cysteines can be cross-linked using arylation reactions with perfluoroaryl groups [49] or using alkylation reactions with bis-bromomethyl or tris-bromomethyl linkers [50,51]. In all cases of α-helix stabilization, an appropriate intramolecular cross-link between cysteine residues promotes helix-nucleating hydrogen-bonding patterns, stabilizing the overall α-helical structure. An advantage of these cyclic peptides is that they are generally more resistant to proteolytic degradation [20,52,53,54]. Some of the cyclic peptides also have increased cytosolic penetration compared to linear peptides [8,53,55,56]. 

DeGrado, Greenbaum, and co-workers have used thiol bis-alkylation to induce α-helical structure in a peptide inhibitor of the protease calpain [50]. The cysteine residues to be cross-linked were placed at i and (i + 4) positions within a model peptide with moderate helicity. Twenty-four different linkers were screened, and it was found that the linker dibromo-m-xylene showed the highest increase in helical structure [50]. Similarly, Muppidi et al., designed stapled helices containing cysteine residues at i and (i + 7) positions, using longer linkers like 4,4′-bis-bromomethyl-biphenyl and 6,6′-bis-bromomethyl-[3,3′] bipyridine [55] yielding a series of BH3-peptide-derived ligands for MCL-1 with increased helicity, bioactivity and even cell permeability. Cysteine residues placed at relative positions from (i, i + 3) to (i, i + 10) can be readily cyclized using the dithiol bis-alkylation reaction. Different linkers like xylene-based linkers, 2,6-bis(bromomethyl) pyridine, biphenyl and napthyl linkers can be used for the stapling of two cysteines with dithiol bis-alkylation. Other thiol-containing amino acids like l- and d-cysteine, l- and d-homocysteine, and l- and d-penicillamine also undergo bis-alkylation with similar efficiency [57]. 

### 2.5. Hydrogen Bond Surrogate Derived α-Helices

Another approach that is gradually gaining eminence is the hydrogen bond surrogate (HBS) approach. The α-helix is characterized by a 13-membered intramolecular hydrogen bond between the C=O of the ith and the NH of the (i + 4)th amino acid residues. The HBS strategy stabilizes the α-helices by replacing one of the main-chain intramolecular hydrogen bonds with a covalent linkage. Cabezas and Satterthwait have proposed a hydrazone bridge as a mimic for the hydrogen bond [58], while Arora and co-workers utilized a carbon-carbon bond prepared by a ring closing metathesis (RCM) reaction [59]. Using the HBS strategy, peptide mimics have been developed, which can target gp41-mediated HIV-1 fusion in cell culture, highlighting the potential of these artificial helices as inhibitors of chosen protein-protein interactions in complex settings [60]. The advantage of the main chain hydrogen bond surrogate strategy over side-chain cross-linking strategies is that the placement of the cross-link on the inside of the helix does not block the solvent-exposed amino acid side chains which act as molecular recognition surfaces of the molecule. This allows HBS helices to target tight binding pockets on proteins [61]. Also, as expected from their conformational stability, HBS helices are significantly more resistant to proteases than their unconstrained counterparts [60,61].

### 2.6. β Peptides

A new emerging class of peptides now being used as mimics of helical peptides is the β-peptides. These peptides consist of proteinogenic β-amino acids. One of the first β-peptidic mimicries of an α-helix was the design and synthesis of a β-nonapeptide which existed as a 3_14_-helix and was shown to inhibit the lipid transport protein SR-B1 [30]. Gellman’s group has extensively studied the introduction of β-peptides into peptide backbones to determine the effects of backbone modification [62,63]. An advantage of this approach is that the side chains are not blocked or removed by a tethering functional group. Several α/β sequences have been studied (αβαβ, ααβαααβ, ααβααβ, αααβαααβ, etc.) [64]. Short β-peptides have been designed and synthesized which can adopt a helical secondary structure in water when stabilized by salt bridges or macrodipole effects [65,66,67]. Over the last few years, many helical β-peptides have been reported to bind diverse protein targets like somatostatin reporters, HDM2 and viral fusion proteins [68,69,70]. Recently, another new β-peptide has been reported to inhibit HDMX, which is another key therapeutic target for activation of p53 in tumors [71].

## 3. Constrained-Helix-Based PPI Inhibitors

The design of peptides for the inhibition of specific PPIs is based on the nature and structure of the interacting surface of two or more proteins. If the proteins interact through helical segments, a short peptide analog of the interacting helix can sometimes function as an inhibitor of the PPI. This approach has been widely used, and some examples are given below. 

### 3.1. BCL-2 Family Proteins

BCL-2 is the founding member of a protein family composed of pro- and anti-apoptotic molecules that provide an essential control point in apoptosis [4,72,73]. Walensky and his group did extensive work in this area using stapled peptides, blazing a path to modulate the BCL-2 pathway. They produced a library of hydrocarbon-stapled peptides that mimic the death domain (BH3) of the pro-apoptotic BCL-2 family member. These stapled peptides, called “stabilized α-helix of BCL-2 domains” (SAHBs), are helical, protease-resistant and cell-permeable molecules that bind to multi-domain BCL-2 member pockets, having increased affinity and desired cellular effects [44]. 

### 3.2. Inhibition of p53 Binding with MDM2/MDMX

p53, the product of the TP53 gene, is a human transcription factor and a tumor suppressor that induces cell-cycle arrest and apoptosis upon cellular stress and DNA damage and thereby playing a crucial role to protect cells from potential malignant transformation. Over-expression of MDM2, an antagonist of p53, or deletion or mutation of the TP53 gene inactivates p53, a common phenomenon in human cancers [74,75]. MDM2 negatively regulates p53 by direct binding. The binding of MDM2 with p53 masks the transactivation domain of p53, impairing the nuclear import of the p53 protein [76]. Also, MDM2 is responsible for ubiquitination and proteasomal degradation of the p53 protein [77]. MDMX, another negative regulator, possesses a similar p53-binding activity and inhibits p53 transcriptional activity [78,79]. The natively unfolded transactivation domain of p53 [80,81] forms a helix when it binds MDM2 [82]. Several groups have used stapled peptides against MDM2 either mimicking the native p53 binding sequence, or non-native sequences, derived from phage display experiments [5,83]. Thean et al., designed and synthesized a library of stapled peptides called sMTIDE-02, targeting p53/MDM2 interaction. However, due to cytotoxicity and inadequate potency to activate p53 in the presence of serum, a significant proportion of this library was further chemically modified. The newly synthesized peptides were named VIP-82 [84]. These chemical modifications not only made the new library of peptides to be less cytotoxic but also provided higher solubility. Chang et al., discovered a highly potent and specific stapled peptide named as ATSP-7041 which acts as a dual inhibitor of MDM2/MDMX. They have shown that these peptides effectively suppress the growth of human tumors in which p53 is inactivated due to MDM2/MDMX over-expression [8].

### 3.3. c-FOS and c-JUN

Rao et al. designed and synthesized a series of Jun-based short helical peptides incorporating unnatural amino acids. These peptides are stable and resistant to proteolytic degradation in serum. These peptides bind with high affinity and specificity to c-Fos, which is a key component of the oncogenic transcriptional regulator Activator Protein-1 (AP-1). Thus, the peptides competitively inhibit the c-Jun–c-Fos coiled-coil interaction [85]. Baxter et al., synthesized a series of helical peptides mimicking c-Fos that bind to Jun with high affinity. They also modified the peptide sequence by attaching a cell penetrating peptide (TAT-48–57) and a nuclear localization signal (SV40). This modification of peptides promotes cellular uptake and nuclear localization, which ensure the inhibition of the cell proliferation [86].

## 4. Synthetic Transcription Factors from Helical Peptides

Over the last several decades, it has become clear that aberrant gene expression is either the primary cause, or a major component, of the morbidity and mortality of many diseases [87]. So far, most of the attempts to regulate gene expression have focused on upstream regulators of gene expression, such as kinases involved in signaling [88]. The direct control of gene expression by targeting the transcription factors, or their binding sites, by synthetic molecules has rarely been achieved in practice. One of the main reasons is the limited variation of the B-DNA structure and conformation, making it difficult to discover or develop molecules with the desired sequence specificity. The first major attempt was made by Dervan and co-workers, who developed polyamides consisting of pyrrole and imidazole units [89]. They developed rules for designing pyrrole-imidazole copolymers for a defined DNA sequence [90]. However, to our knowledge, notwithstanding decades of work, this class of molecules has failed to reach the clinic [91]. Peptides offer significant advantages as synthetic transcription factors; they are protein-like. The peptide therapeutics market is large and growing, making the translation pipeline easier to define and manage [92]. They can also be produced in large quantities with high purity, with relative ease. Peptides are recognized for being highly selective and efficacious and, at the same time, relatively safe and well tolerated and biodegradable.

Many natural transcription factors use the α-helix as one of the elements for the readout of the DNA sequence [93]. In a large majority of the cases, unlike polyamides, these helices interact through the information-rich major groove, raising the possibility of attaining higher specificities compared to the minor groove binders. Thus, constrained α-helical peptides may be considered suitable building blocks for synthetic transcription factors. To our knowledge, the first attempt to use a peptide to mimic a transcription factor came through the work of Kim and his co-workers [94]. A dimeric di-sulfide linked peptide mimic of transcription factor GCN4 containing the basic region of bZIP motif was synthesized, which bound to the target site specifically in vitro, but only at 4 °C. That peptide also adopted α-helical structure when bound to the DNA [94]. In 1999, Schepartz and coworkers used a mini-protein to create a synthetic DNA-binding protein by grafting the DNA-interacting residues of a bZIP protein [95]. The mini protein itself provided the conformational constraint. Pioneering studies by Mascarenas and his co-workers established the notion that engineered peptides can be useful mimics of transcription factors [96,97,98]. The first use of constrained α-helices for sequence-specific DNA binding came through the work of Woolley and a co-worker, in the context of photo-switchable DNA-binding helices [99,100,101]. Later, a helix, constrained through stapling, was used by Kajino et al., to bind a specific DNA sequence with high affinity [102]. In 2015, a single and stable α-helical peptide was designed via peptide stapling as a DNA-binding element. Two optimally positioned cysteine residues in the monomeric DNA-binding domain were linked. This resulted in enhanced helicity, DNA binding ability, and enhanced cellular uptake [103]. However, in none of these studies was gene regulation by these agents studied inside a cell in any significant manner. 

**Figure 2:**
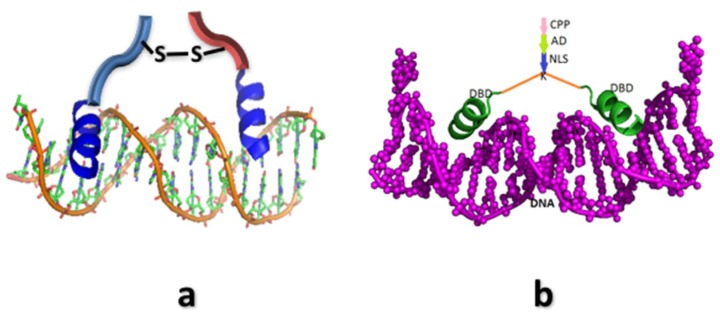
Cartoon diagram of structure of (**a**) Synthetic Transcription Factor (STF) mimicking the λ-Cro; Bright blue part is the helical part of the construct, while the linker regions are represented in light blue and red colours. (**b**) STF mimicking the λ-Cro but carrying mammalian NLS, CPP, and Activation Domain (AD). Orange chain denotes the linker.

Our group developed α-amino isobutyric acid (Aib) substituted conformationally-constrained helices as building blocks for constructing Protein-Protein interaction [27,104,105] and Protein-DNA interaction inhibitors. Tagged with the CPP (cell penetrating sequence) and the NLS (nuclear localization signal), the peptide constructs targeted against specific DNA sequence—henceforth, called synthetic transcription factors (STF)—accumulate in the nucleus and regulate the expressions of desired genes. Our initial attempt was to develop a specific DNA-binding peptide mimic of λ-Cro, a phage DNA-binding protein [106,107]. The helix building block was prepared by substituting a few non-interacting amino acids with α-amino isobutyric acid (Aib) on the non-interacting surface [27]. A suitably cross-linked dimeric peptide was created by cross-linking the helices (Figure 2). The dimeric construct bound O_R_3, a Cro binding site in the bacteriophage λ genome, with good affinity and single basepair discrimination specificity. When internalized into *E. coli* cells which harbored a plasmid containing the GFP gene under the control of λ-P_R_ promoter (containing O_R_1, O_R_2, and O_R_3 operator sites), significant repression of the GFP expression was observed [106]. A more nuanced design of the STF was created to explore whether up-regulation of a target gene can be achieved in a mammalian cell. For this experiment, a variant of the peptide was tagged with NLS and CPP, and conjugated to a known eukaryotic activation domain, the Kix binding peptide (KBP) (Figure 2). When applied to a mammalian cell in which a luciferase reporter gene was put under the control of several of the STFs target sites, the transcription of the luciferase gene was significantly up-regulated [107].

**Figure 3:**
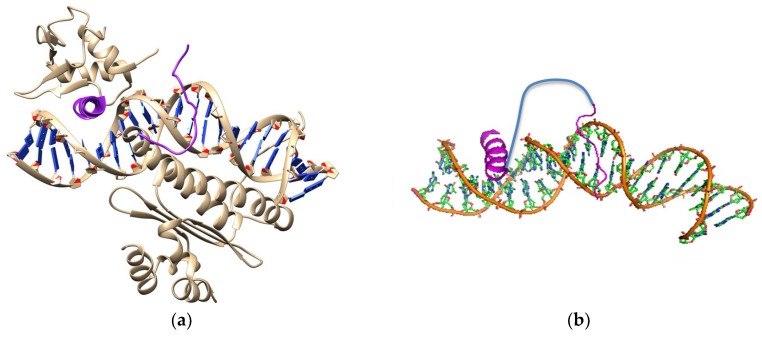
(**a**) Structure of SAP-1/SRF co-complex with target DNA (pdb1K6O); SAP-1 is a paralog of ELK-1; (**b**) Cartoon depiction of the synthetic transcription factor targeted against both ELK-1 and SRF sites. Magenta helix is the DNA major groove binding helix from Elk-1 protein; Magenta chain is a loop derived from SRF protein and binds to the DNA minor groove; Blue chain is the linker.

Another attempt was made to control the expression of c-FOS gene in a RAS mutant cell line (93). The aberrantly activated EGFR-RAS-MAP kinase pathway bearing an oncogenic mutant RAS protein is the driver of about 20% of cancers [108]. One of the important end-points of the RAS-MAP (Mitogen-activated protein kinase) kinase pathway is the ETS (E-26 transformation-specific family of proteins) family transcription factor, ELK-1. Gene regulatory action of ELK-1 on the c-FOS promoter occurs upon simultaneous and cooperative binding of the serum response factor (SRF) to adjacent sites of the c-FOS promoter [109,110]. A modular approach was adopted for the design of this STF. An Aib-containing helically constrained peptide encompassing the DNA-interacting segments of ELK-1 (helix), and the proximal minor groove binding part of SRF was linked by a suitable linker sequence (Figure 3). The designed peptide showed good affinity and single basepair discrimination specificity towards the target DNA site. To test the efficacy of this peptide in the lung adenocarcinoma cell-line that bears an oncogenic mutant RAS allele, the peptide was tagged with a CPP and a NLS to deliver it inside the nucleus. The designed peptide specifically down-regulated expression of the c-FOS gene significantly by displacing the original transcription factor complex from its promoter region [108]. 

**Figure 4:**
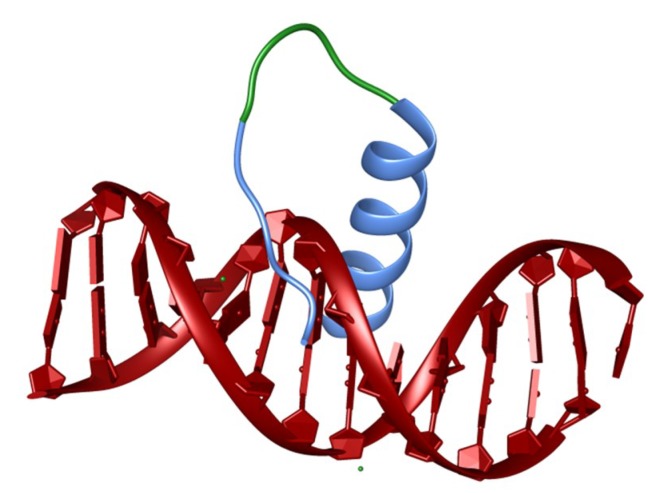
Structure of the BP-1 homeodomain mimicking STF. The blue segments of the STF were derived from the homeodomain and the green segment was the designed linker.

In a recent study, our group reported the construction of a homeodomain-mimicking STF that contains two DNA-recognition elements present in the homeodomain: The recognition helix and the *N*-terminal arm [111] (Figure 4). The recognition helix was conformationally-constrained by the suitable incorporation of Aib at non-interacting positions. The mimicked transcription factor was homeodomain-containing BP1, a well-known repressor of the β-globin gene. This construct, like that of the wild-type homeodomain, was capable of discriminating basepair substitutions in the core DNA sequence. When conjugated to the CPP and the NLS sequences, this peptide up-regulated β- and γ-globin genes in the CD34^+^ human hematopoietic stem and progenitor cells derived from human cord blood. The use of cell-permeable constrained helical peptides that interfere with and inhibit protein-DNA interactions in vivo looks promising for the future therapeutic purpose. 

Recently, Payne et al. have reported the design and synthesis of a conformationally-constrained hydrocarbon-stapled peptide based on a DNA-interacting α-helix from the leucine zipper motif of the σ^54^ subunit of bacterial RNA polymerase. The peptide was capable of penetrating the membrane of the gram-negative bacteria through diffusion. It then binds to the target promoter sequence, thereby inhibiting interaction between endogenous σ^54^ and its target DNA sequence and repressing the σ^54^ mediated gene expression in bacteria. This peptide can be further developed for the treatment of infections by gram-negative pathogens [112]. 

## 5. Strategies for Targeting of Constrained Helical Peptides 

In the past, peptides were assumed to be inappropriate as drug candidates, particularly against intracellular targets, when compared to small organic molecules due to unfavorable pharmacokinetic parameters and lack of cell penetration ability. In general, main limitations which inhibit use of unmodified peptides as a therapeutic agent are poor ability to cross membranes because of their polar nature, a short half-life because of their rapid degradation by the proteolytic enzymes of blood plasma, rapid plasma clearance due to renal glomerular filtration, and low oral bioavailability [113,114]. In spite of the above-mentioned limitations, peptides are preferred over small organic molecules as potential therapeutic agents, due to their higher affinity/specificity towards target binding and lower toxicity profiles in general. Increasingly, peptides are being accepted as good drug candidates, as the limitations mentioned above are being overcome [90]. We will not discuss the issue of oral bioavailability as peptides are rarely administered through the oral route. 

### 5.1. Cell Penetration

Targeting helical peptides to intracellular proteins or DNA, either ex vivo or in vivo, requires translocation into the live cell. The plasma membrane of the cell consists of a hydrophobic lipid bilayer, making it difficult for hydrophilic compounds, such as an average peptide, to cross the membrane unless they avail existing cellular mechanisms of entry. One such general mechanism is endocytosis or pinocytosis, which may be availed through conjugation of the desired peptide to a cationic cell-penetrating peptide (CPP). Cell-penetrating ability of certain peptides was first observed in the natural HIV Tat transactivator [115] and the homeodomain *Drosophila melanogaster* transcription factor Antennapedia [116]. Later, some non-natural peptides have been found to be very efficient CPPs [117,118]. We have extensively used a stretch of six d-arginines as CPP—to keep the peptide small—with satisfactory results. The six d-arginines at the *N*-terminus served a dual purpose. It acted as a CPP and being a d-peptide, putatively, prevents exo-peptidase cleavages from the terminus. Cell penetrating peptides have been reviewed many times recently and readers interested may consult some recent reviews [119,120]. 

### 5.2. Plasma Stability

Plasma half-life plays an important role in the efficacy of therapeutic peptides. Unmodified and unconstrained peptides are often rapidly degraded by proteases present in the blood plasma. Chemical modifications and conformational constraints prevent many proteases from rapidly hydrolyzing peptides. The most widely used method to increase the stability of therapeutic peptides is the substitution of natural l-amino acid by non-natural d-amino acid. Examples include replacement of specific glycine residues with d-serine in the bicyclic peptide inhibitor of the cancer-related protease urokinase plasminogen activator. This led to improvement of not only the potency by 1.8 times but also increasing the stability by four-fold in the mouse plasma [121]. Similarly, l-arginine containing vasopressin has a half-life of 10–35 min in humans, whereas the corresponding d-Arg-containing analog, desmopressin, has a half-life of 3.7 h. For constrained helices, there is ample evidence that conformational constraints themselves reduce proteolytic cleavage, making additional modifications often unnecessary.

### 5.3. Pharmacokinetics

Rapid renal clearance of peptides is assumed to be a major problem that must be overcome before the development of peptide can be undertaken as a successful therapeutic agent [122]. Hydrophilic peptides having molecular weight 2–25 KDa are easily absorbed by the renal tubule and therefore are susceptible to frequent filtration through the glomeruli of the kidney having a pore size of ~8 nm. Endocytosis and degradation by the proteasome and the liver are the other minor route of peptide clearance. The comparative study of systemic and renal clearance in an animal model has explained that the renal clearance is the major elimination pathway. The common strategies have been used to inhibit renal clearance:

Glomerular filtration can be reduced by covalently linking albumin binding small molecules to peptides. The indirect interaction of the peptide with albumin through the highly bound small molecule improves proteolytic stability and prolongs the half-life [123]. For example, when an albumin-binding peptide was linked to a bicyclic peptide, it became resistant to proteolysis and led to a 50-fold increase in half-life [124]. Renal clearance of peptide is also reduced when the molecular weight and the hydrodynamic volume of the peptide are increased. This can be done by conjugation of peptides to large synthetic molecules, carbohydrates or natural polymers. The most commonly used polymers for this purpose are polyethylene glycols (PEG), hydroxyethyl starch (HES) and polysialic acid (PSA). 

### 5.4. Immunogenicity

The tendency of a therapeutic agent to evoke an unwanted immune response is referred to as immunogenicity. It is an important concern for protein/peptide therapeutics and often results in the formation of anti-drug antibodies (ADA) after repeated and chronic administration [125]. ADA formation may not only modify the biological activity of the therapeutic agent but may also influence its pharmacokinetic profile. Small peptides of molecular weight less than 4 kDa are generally believed to be poor immunogens. However, due to the complexity of the human immune response, exceptions have been observed [126]. One of the most recent example is that of taspoglutide, a 30-mer chemically modified peptide, the first once-a-week GLP-1 analog based on a human sequence. As a result of a relatively uncommon but serious hypersensivity reaction that could be related to ADA formation, its development was discontinued [127]. The administration pathway may affect immunogenicity [128]. The higher incidence of immunogenic reaction is often associated with the subcutaneous route of administration in comparison to that of the intramuscular or the intravenous route [128]. This may be due to the increased likelihood of formation of protein aggregates when administered by the subcutaneous route, a factor known to increase immunogenicity [128]. The immunogenicity of peptides can be avoided by avoiding potential antigenic sequences in the primary structure and structural modifications, such as, glycosylation or PEGylation, as these can shield antigenic determinants on the drug through steric hindrance from detection by the immune system [125]. For example, immunogenicity of a 17-residue peptide from wasp venom (Vespulakinin, VSK-1) was dramatically decreased when carbohydrate was attached to it [129]. Another example is PEGylated TPO mimetic peptide caused no immune mediated lesions in mice, but the recombinant human TPO (thrombopoietin) suppressed megakaryocytopoiesis and caused B-lymphocyte hyperplasia in lymphoid tissue in mice, consistent with antigenic stimulation [130]. 

Constrained helices may be less susceptible to elicit an immune response as they are unlikely to bind to MHCII molecules as the MHCII displayed antigens are generally in stretched conformation. Our experiments seem to bear that out as some constrained helices failed to elicit a strong immune response [104]. The immunogenicity of helical peptides, if they occur at all, can be further avoided by avoiding antigenic sequences in the amino acid sequence and structural modification such glycosylation or PEGylation as these can shield antigenic determinants on the drug through steric hindrance from detection by the immune system [125]. 

## 6. Conclusions

The development of many methods of conformationally constraining short peptides to helical conformation has opened up new avenues for inhibiting a large class of protein-protein and protein-DNA interactions, which many considered as “undruggable”. With the advent of new techniques for cell penetration, the improvement of ADME parameters and large scale of production of synthetic peptides, the prospect of intracellular targeted, conformationally-constrained helical peptides entering clinical use looks good.
